# Achieving and maintaining superior postoperative outcomes with an optimized recovery after thoracic surgery protocol

**DOI:** 10.1016/j.xjon.2025.08.018

**Published:** 2025-09-24

**Authors:** Dao Minh Nguyen, Ahmed Alnajar, Mehmed Akcin, Nestor Villamizar

**Affiliations:** Division of Thoracic and Foregut Surgery, The DeWitt Daughtry Family Department of Surgery, University of Miami, Miami, Fla

**Keywords:** enhanced recovery after thoracic surgery, robotic anatomic resections, postoperative pain, opioid use

## Abstract

**Objective:**

Protocol optimization to improve postoperative outcomes is a key component of enhanced recovery after surgery program. The aim of this study is to determine the long-term impact of protocol optimization on postoperative outcomes of robotic pulmonary anatomic resections.

**Methods:**

A retrospective analysis of a prospectively maintained database was performed. All elective robotic anatomic pulmonary resections between February 1, 2018, and December 31, 2023, were included and stratified into original Enhanced Recovery After Thoracic Surgery (ERATS) - group A (February 1, 2018, to December 31, 2019, n = 184) and optimized ERATS - group B (January 1, 2020, to December 31, 2023, n = 557). Propensity-score matching yielded highly comparable cohorts for outcome analysis. Data included demographics, operative details, postoperative outcomes (hospital length of stay [LOS], postoperative opioid use, 30-day complications), patient-reported subjective pain, and cost-savings resulting from reducing observed LOS versus expected LOS on the basis of Medicare Severity Diagnosis-Related Group classification and estimated institution per-diem cost of $1100.00.

**Results:**

Of the 732 eligible patients, 499 were matched with 173 to group A and 326 group B. Protocol optimization was associated with significant improvement of postoperative outcomes: shorter LOS, greater percentages of postoperative day 1 discharge, substantial less opioid requirements with increasing number of opioid-free postdischarges, and greater cost-savings either collectively or per individual DRGs attributable to the reduction of LOS. Annual auditing of the optimized ERATS cohort demonstrated consistent improvements in key metrics over time.

**Conclusions:**

Successful ERATS protocol optimization and maintenance was associated with durable and significantly superior postoperative outcomes along with greater cost-savings attributable to a reduction of LOS in patients undergoing elective robotic pulmonary anatomic resections.

**Figure:**
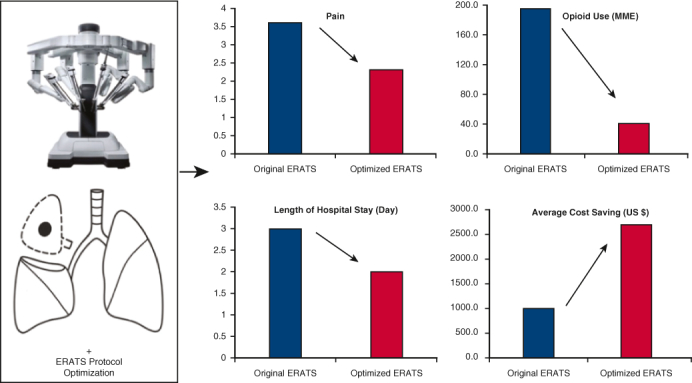
Achieving and maintaining superior postoperative outcomes by ERATS protocol optimization.

**Central Message**

It is not only essential to implement a functional ERATS protocol but also important to optimize and to monitor its performance to ensure consistent achievement of superior postoperative outcomes.

**Perspective**

Achieving and maintaining superior postoperative outcome metrics via process optimization of an already functional enhanced recovery protocol after robotic pulmonary anatomic resection particularly with almost pain-free and opioid-free discharges augments patient satisfaction and contributes to the fight against opioid abuse epidemic.

The Enhanced Recovery After Thoracic Surgery (ERATS) protocol, as previously reviewed,^1^^,^^2^ has gained significant traction in being a standard of perioperative care for patients undergoing thoracic surgical procedures worldwide. Successful implementation of the ERATS protocol is associated with significant reduction of opioid use while achieving effective mitigation of acute somatic incisional pain, decrease of postoperative complications, shortened length of stay (LOS) in the hospital, and maximal patient comfort and satisfaction.^3^, ^4^, ^5^, ^6^ Whereas many components of the ERATS protocol act synergistically to yield the observed salutary outcomes, effective pain control likely contributes most directly to the reduction of postoperative complications and LOS.

An enhanced recovery protocol emphasizes the importance of regular auditing of protocol performance by critical reviewing outcomes and making modifications for improvements.^7^ Our group implemented ERATS in February 2018 and demonstrated a significant reduction of postoperative acute pain and in-hospital opioid use compared with pre-ERATS historical controls.^3^^,^^4^ We reviewed the performance of our initial protocol and made concrete changes, as outlined in the Methods section and Table E1 in late 2019, aiming for further reduction of opioid use and postoperative pain.^8^ Our current ERATS protocol is the optimized version that was implemented on January 1, 2020, without added modifications. In addition to further reduction of total postoperative opioid use and acute pain levels, we have observed increasing numbers of patients discharged home without opioids as well as increasing incidences of discharge on postoperative day (POD) 1 in patients undergoing robotic procedures and receiving the optimized ERATS protocol compared with those managed by the original protocol.^9^^,^^10^ Moreover, a significant reduction of total hospital cost associated with enhanced recovery protocol has been reported for thoracotomy, video-assisted thoracoscopy, and robotic approaches.^7^^,^^11^ Patient-reported pain levels, opioid use, LOS and 30-day postoperative complications, and cost-savings are readily measurable and reliable metrics of ERATS outcomes and can be used for longitudinal comparative analysis of protocol performance over time.

We set out in this retrospective study to comprehensively review relevant postoperative outcomes: LOS, complications, pain levels, opioid use, and cost-savings associated with discharging patient sooner than expected LOS using MS-DRG (Medicare Severity Diagnosis-Related Group) classification of patients undergoing robotic anatomic pulmonary resection receiving perioperative care with either the original or the optimized ERATS protocol over the last 5 years. The primary objective of this study was to define the enduring impact of protocol optimization on improving postoperative outcomes (LOS, pain level, and opioid use) over those of the original protocol. The secondary objective was to determine cost-savings associated with shortening LOS by ERATS protocols.

## Methods

### Patient Population

A retrospective analysis of data extracted from our prospectively maintained thoracic surgery database was performed following institutional review board approval with a waiver of patient consent requirement (institutional review board number 20180827; last reviewed and approved December 20, 2024). All adult patients (>18 year old) undergoing elective robotic anatomic pulmonary resections (segmentectomy, lobectomy, bilobectomy, pneumonectomy with intrathoracic lymphadenectomy for pulmonary malignancy) by 2 senior surgeons (D.M.N., N.V.) from February 1, 2018, to December 31, 2023, were reviewed. The following patients were reported but not included in pain and opioid use analysis: those who stayed on endotracheal intubation/mechanical ventilation, those admitted to the intensive care unit, and those who underwent conversion to open thoracotomy or experienced in-hospital death. We implemented our original ERATS protocol on February 1, 2018, for all patients undergoing thoracic surgery. A detailed description of protocol development, implementation, and early results has been previously reported.^3^^,^^4^ The main feature of pain management was the intrathoracic infiltration of 9 intercostal spaces under direct vision for intercostal nerve blocks using 20 mL of liposomal bupivacaine (LipoB; Exparel, Pacira Pharmaceutical, Inc) diluted in 30 mL of normal saline and multimodal scheduled nonopioid and pro re nata (PRN) opioid administrations. The following modifications were implemented on January 1, 2020: using 30 mL of 0.25% bupivacaine as diluent instead of saline, switching tramadol from scheduled to PRN dosing, removal of the bladder catheter on morning rounds on POD 1, and early removal of the chest tube (Table E1).^8^ Patients were stratified to original ERATS cohort (February 1, 2018, to December 31, 2019—group A) and the optimized ERATS cohort (January 1, 2020, to December 31, 2023—group B), with group A being the historic control group of this study. Propensity-score matching (PSM) was conducted to achieve harmonization of the 2 groups for more valid comparative analysis. The study was conducted and reported in concordance with the Strengthening the Reporting of Observational studies in Epidemiology guidelines.^12^

### Data Source and Attributes

The thoracic surgery database prospectively collects from the hospital electronic medical records (EPIC) detailed clinical parameters, including but not limiting to patient demographics, operative details, pathologic diagnoses, tumor-node-metastasis (ie, TNM) staging for primary lung cancer, 30-day postoperative complications (Clavien-Dindo classification), LOS and readmission, daily pain scores recorded by nursing staff as previously described,^8^^,^^10^ and in-hospital analgesics dispensed (schedule II opioids oxycodone, hydromorphone, morphine, and fentanyl and schedule IV opioid tramadol; nonopioid analgesics: acetaminophen, gabapentin, and nonsteroidal anti-inflammatory drugs—ketorolac, ibuprofen or celecoxib). The quantities of opioids dispensed are expressed as oral morphine milligram equivalents (MMEs). Information regarding postdischarge readmissions, either to our hospital or to another health care facility, was obtained from EPIC and via postdischarge telephone follow-ups and clinic visits. Fillings of opioid prescriptions within 30 days of discharge were derived from the Florida's Prescription Drug Monitoring Program database. Data are collected by our nurse practitioners and research assistant. The database is monthly audited for accuracy by one surgical faculty member (D.M.N.).

### Cost-Saving Analysis

The MS-DRG coding for each patient was provided by the hospital billing department. Approximately 95% of our patients were assigned 163, 164, or 165 MS-DRG codes. The expected arithmetic means of LOS for each MS-DRG was obtained via querying the CMS.gov website (last access April 2025) for fiscal years 2020 to 2022 (https://www.cms.gov/medicare/payment/prospective-payment-systems/acute-inpatient-pps/fy-2022-ipps-final-rule-home-page). Variances between observed LOS and expected MS-DRG−derived LOS were calculated and rounded up/down to nearest integers (full day of hospital stay). The per-diem cost of admission to acute care medical/surgical unit of our hospital was approximately $1100 (FY 2020). Negative variance of observed LOS – expected LOS × $1100 denotes cost-savings per case. Cost analysis was performed on the entire population of each cohort or stratified to each MS-DRG subgroup to determine the effect of MS-DRG classification on the magnitude of cost-savings.

### Propensity Score Matching

PSM was performed to create matched group A and group B cohorts with reduced confounders. A logistic regression model was specified with the treatment group as the outcome and the following covariates: age, female sex, body mass index, smoking status, coronary artery disease, renal failure, diabetes, hypertension, forced expiratory volume in 1 second percent predicted, and diffusing capacity of the lung for carbon monoxide percent predicted. The matching was performed using nearest-neighbor matching without replacement at a 2:1 ratio. A caliper width of 0.05 was applied to limit poor matches. The MatchIt package in R was used to implement the matching. Covariate balance was assessed using standardized mean differences and empirical cumulative distribution functions. Matching aimed to ensure exchangeability between the cohorts (Table E2). Matched samples were then used in subsequent analyses of pain, opioid use and cost-saving outcomes.

### Statistical Analysis

Demographics, operative/pathologic data, incidences of complications, postoperative clinical outcomes (pain levels, LOS, opioid use), and cost reduction were summarized using descriptive statistics (frequencies and percentages; means and standard deviations for normally distributed variables with normality determined by the Kolmogorov-Smirnov test; medians and interquartile ranges Q1-Q3) and compared using the Fisher exact test for categorical variables and Mann-Whitney *U* test or Student *t* test for continuous variables where appropriate. Statistical analysis was performed using GraphPad Prism (version 10.4.2).

## Results

A total of 741 patients fulfilled the inclusion criteria for this study (184 of original ERATS cohort—group A and 557 of optimized EATS—group B). Of 732 patients eligible for postoperative pain and opioid use analysis subjected to PSM, 499 were matched with 173 in group A and 326 in group B. Demographic data, operative details, pathologic results, and postoperative outcomes of PSM patient populations are shown in Tables 1 and 2. Even before PSM, baseline characteristics of both groups were very comparable (Table E3). Protocol optimization was associated with statistically significant reduction of LOS and more early discharges on POD 1 as well as pain levels on POD 1 and on day of discharge but not on day of the operation (POD 0). More importantly, drastic reduction of in-hospital opioid use and greater percentage of patients who never used opioids within 30 days after discharge were observed in group B (median MME, 24.0 [9.5-48.2] vs 40.0 [25.5-68.0], *P* < .0001, and 63.8% vs 12.1%, *P* < .0001 group B vs group A, respectively) (Table 2). This translated to an overall reduction of postoperative total opioid requirement after ERATS optimization (median MME, 0.0 [0.0- 60.0] vs 150.0 [60.0-250.0], *P* < .0001). There was no difference in percentages of patients taking acetaminophen, gabapentin, and nonsteroidal anti-inflammatory drugs in both cohorts (data not shown). Profound postoperative outcome improvements by protocol optimization in PSM cohorts were readily seen in prematched populations (Table E4).

**Table 1:** Demographics and clinical characteristics of PSM original ERATS (group A) and optimized ERATS (group B)

Demographics	Group A (n = 173)	Group B (n = 326)	*P* value
Gender (F:M)	103:70	196:130	.63
Age, y	69.0 [62.0-75.0]	68.5 [61.0-75.0]	.92
Race/ethnicity			
White/Black/Asian + other	154 (89.0%)/12 (6.9%)/7 (4.0%)	299 (91.7%)/17 (5.2%)/10 (3.1%)	.51
Hispanic/non-Hispanic	76 (43.9%)/97 (56.1%)	149 (45.7%)/177 (54.3%)	.7768
BMI, kg/m^2^	26.6 [24-30.4]	27.0 [23.7 -30.0]	.56
FEV1 (% of normal)	87.0 [75.0-97.2]	86.0 [74.0-99.0]	.49
DLCO (% of normal)	79.0 [69.0-92.0]	78.0 [67.0-90.0]	.96
**Intraoperative characteristics**			
Operating time, min, median [IQR]	194.0 [170.0-219.0]	183.0 [152.0-229.2]	**.019**
Estimated blood loss, mL	50.0 [50.0-100.0]	50.0 [30.0-100.0]	**.008**
Conversion to open	0	0	
Lobectomy	130 (75.1%)	226 (69.3%)	.178
Segmentectomy	43 (24.8%)	100 (36.7%)	
Diagnosis, n (%)			
Benign	5 (2.9%)	14 (4.3%)	.624
Malignant	168 (97.1%)	312 (95.7%)	
Secondary	12 (7.2%)	17 (5.4%)	
Primary	156 (92.8%)	295 (94.6%)	
Stage 0-1	127 (81.4%)	216 (73.2%)	.063
Stage 2-4	29 (18.6%)	79 (26.8%)	

Values in bold indicate statistical significance. *F*, Female; *M*, male; *PSM*, propensity score matching; *BMI*, body mass index; *FEV1*, forced expiratory volume in 1 second; *DLCO*, diffusing capacity of the lung for carbon monoxide; *IQR*, interquartile range.

**Table 2:** Postoperative outcomes of PSM populations of original ERATS (group A) and optimized ERATS (group B)

	Group A (n = 173)	Group B (n = 326)	*P* value
Observed LOS, median [IQR]/mean ± SD	**3.0 [2.0-3.2]/3.4 ± 2.6**	**2.0 [1.0-3.0]/2.8 ± 2.7**	**<.0001**
Discharge on POD 1, n (%)	**9 (5.2%)**	**103 (31.6%)**	**<.0001**
Readmission, n (%)	7 (4.0%)	12 (3.7%)	.811
30-d complications (Clavien-Dindo), n (%)			
0	139 (80.3%)	279 (85.6%)	.160
1-2	19 (11.0%)	29 (8.89%)	1-2 vs 2-4: *P* = .65
3-4	15 (8.7%)	18 (5.49%)	
5 (mortality)	0	0	
Duration of chest tube use, h, median [IQR]	**45.0 [28.0-65.7]**	**26.0 [21.5-47.5]**	**<.0001**
Pain (0-10 visual analog scale), median [IQR]	n = 173	n = 326	
POD 0	4.8 [3.3-6.0]	4.6 [3.3-5.7]	.190
POD 1	**3.6 [2.0-5.0]**	**2.3 [1.2-3.7]**	**<.0001**
On day of discharge	**2.0 [1.1-3.1]**	1.5 [0.0-2.9]	**.001**
Opioid use, MME, median [IQR]			
Average in-hospital	**21.2 [15.0-29.3]**	**12.4 [5.0-24.2]**	**<.0001**
Total in-hospital	**40.0 [25.0-68.0]**	**26.0 [9.1-52.7]**	**<.0001**
30-d postdischarge	**150.0 [60.0-271.1]**	**0.0 [0.0-60.0]**	**<.0001**
Total postoperative (in-hospital + 30-d postdischarge)	**196.5 [121.0-330.5]**	**41.2 [10.1-101.3]**	**<.0001**
MME0 (no opioid Rx filling ≤30 d postdischarge)	**17 (9.8%)**	204 (62.4%)	**<.0001**

Values in bold indicate statistical significance. *IQR*, Interquartile range; *SD*, standard deviation; *POD*, postoperative day; *MME*, morphine milligram equivalents.

**Figure 1:**
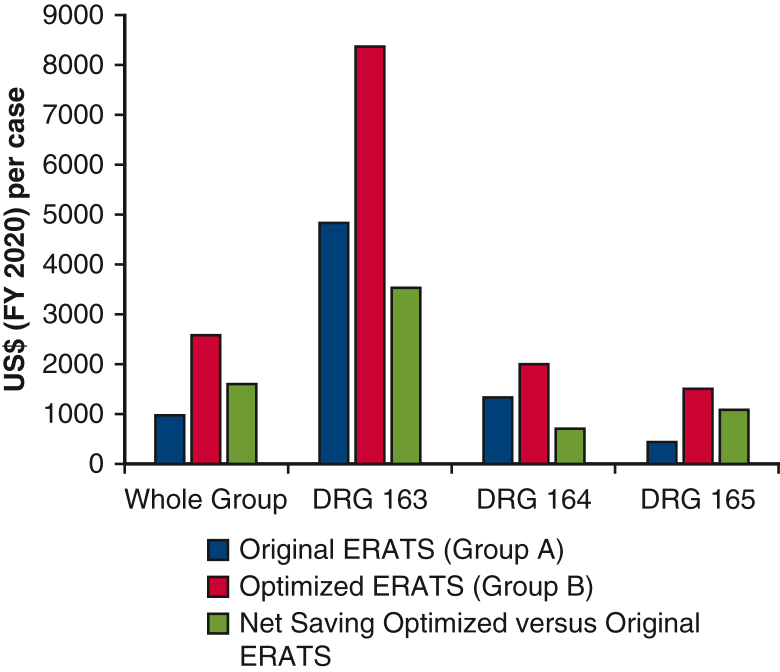
Average per case cost-savings in whole group and in MS-DRG−stratified subgroups in matched populations of original ERATS and optimized ERATS cohorts. *MS-DRG*, Medicare Severity-Diagnosis Related Group; *ERATS*, Enhanced Recovery After Thoracic Surgery.

Table 3 shows detailed analysis of cost-savings in PSM cohorts attributable to LOS variances by shorter observed LOS versus MS-DRG- expected LOS either as whole group or following stratification into 3 major MS-DRG group. Both ERATS protocols were associated with cost-savings but much more pronounced in the optimized ERATS by virtue of shorter LOS. An average cost-savings of about $973.0 per patient of group A and $2578.0 per patient of group B for a net increase of about $1641.0 in cost-savings by protocol optimization (Figure 1). The difference in cost-savings between the 2 cohorts was greatest in MS-DRG 163 followed by 165 then 164 subgroups. All cost-saving differences were highly statistically significant (Mann-Whitney *U* test, *P* < .0001). Significantly more patients in group B were classified in high case-mix indices MS-DRG 163 and 164 compared with group A (163: 10.1% vs 5.8%; 164: 57.0% vs 38.7%, *P* < .0001, Fisher exact test). Identical differential cost-savings between group A and group B were also seen in unmatched cohorts (Table E5).

**Table 3:** Cost-saving analysis of 2 propensity score−matched cohorts

	Group A	Group B	*P* value
**Whole group**	n = 173	n = 326	
Observed LOS, mean ± SD	**3.4 ± 2.6**	**2.8 ± 2.7**	**<.0001**∗
Expected LOS (MS-DRG)	4.5 ± 2.1	5.4 ± 2.5	
Variant expected-observed LOS	**1.07 ± 2.5**	**2.6 ± 3.1**	**<.0001**∗
Cost-savings, mean ± SD/median [IQR]	**972.8 ± 2776.3** **1100.0 [0.0-2200.0]**	**2577.9 ± 3536.6** **2200.0 [1100.0-3300.0]**	**<.0001**†
**DRG 163, n (%)**	**n = 10 (5.8%)**	**n = 33 (10.1%)**	**.0001**‡
Observed LOS, mean ± SD	**7.6 ± 4.7**	**4.4 ± 3.9**	**.025**∗
Expected LOS	11.7	11.7	
Variant expected-observed LOS	**4.4 ± 4.6**	**7.6 ± 3.9**	**.025**∗
Cost-savings, mean ± SD/median [IQR]	**4840.0 ± 5138.6** **5500.0 [1100.0-9625.0]**	**8379.4 ± 4316.0** **9900.0 [7700.0-11,000.0]**	**.0253**†
**DRG 164, n (%)**	n = 67 (38.7%)	n = 186 (57.0%)	
Observed LOS, mean ± SD	3.8 ± 2.7	3.2 ± 2.9	**<.0001**∗
Expected LOS	5.4	5.4	
Variant expected-observed LOS	**1.2 ± 2.7**	**1.8 ± 2.9**	**<.0001**∗
Cost-savings, mean ± SD/median [IQR]	**1329.8 ± 2957.4/2200.0 [1100.0-3300.0]**	**2003.8 ± 3243.8/3300.0 [1100.0-3300.0]**	**<.0001**†
**DRG 165, n (%)**	n = 90 (52.0%)	n = 94 (28.8%)	
Observed LOS, mean ± SD	**2.6 ± 1.3**	**1.6 ± 0.8**	**<.0001**∗
Expected LOS	3.1	3.1	
Variant expected-observed LOS	**0.4 ± 1.3**	**1.4 ± 0.8**	**<.0001**∗
Cost-savings, mean ± SD/median [IQR]	427.8 ± 1425.1/1100.0 [0.0-1100.0]	**1521.3 ± 930.5/2200.0 [1100.0-2200.0]**	**<.0001**†

Values in bold indicate statistical significance. *LOS*, Length of stay; *SD*, standard deviation; *MS-DRG*, Medicare Severity-Diagnosis Related Group; *IQR*, interquartile range.

∗ Student *t* test.

† Mann-Whitney *U* test.

‡ % of DRG 163, 164, 165 between 2 groups: <.0001, Fisher exact test.

**Figure 2:**
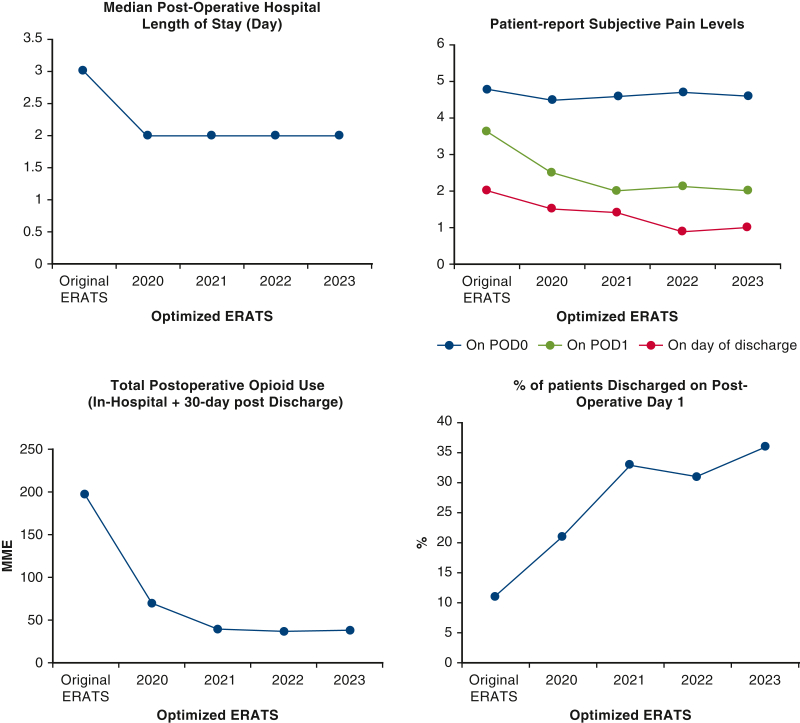

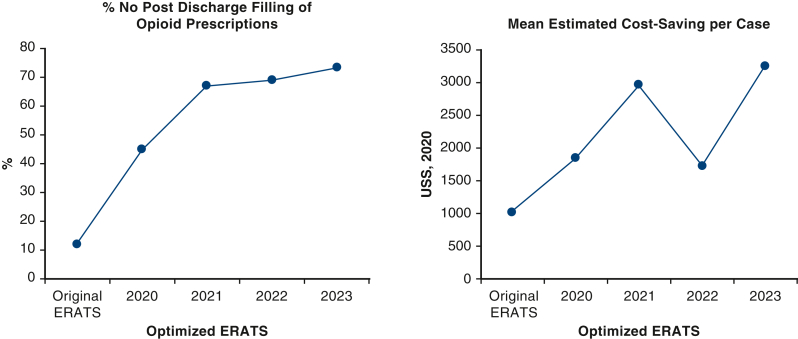
Maintenance of 6 relevant superior outcomes of the matched populations of the optimized ERATS cohort over time compared with the original ERATS cohort. *POD*, Postoperative day; *ERATS*, Enhanced Recovery After Thoracic Surgery.

Time-course analysis of quantifiable postoperative metrics in PSM cohorts showed sustaining superior outcomes in group B compared with the historical control group A (Figure 2). Some metrics such as visual pain scores, LOS, and total postoperative opioid use remain leveled over the 5-year period whereas others (cost reduction, no opioids after discharge, POD 1 discharges) showed improvement over time. Identical findings were seen in unmatched cohorts (data not shown).

## Discussion

We previously reported our initial experience with improvement of commonly reported postoperative outcomes (pain and opioid use) after protocol optimization that was implemented on January 1, 2020.^8^ This current study confirms the sustainability of superior results by protocol optimization not only of the initial standard metrics but also many other postoperative outcomes, only made possible with large cohort of patients and over longer period of time. Most notably are the drastic reduction of postoperative pain and in-hospital as well as postdischarge opioid needs in the optimized ERATS cohort. Substantial percentages of patients were discharged opioid-free for up to 30 days after leaving the hospital. Patients-report subjective pain levels remained low even in the setting of minimal in-hospital opioid use.

Increasing body of literature attests to the role of ERATS implementation on reducing postoperative (schedule II) opioid use and postoperative acute pain (^9^ and references therein). These 2 are inherently connected and targeting pain with nonopioid multimodal analgesia and effective durable regional analgesia with intercostal nerve blocks will lead to less reliance opioids for pain management. Further reduction of pain on POD 1 and later in group B is attributable to many factors, including expedited removal of chest drains and possible greater total amount of bupivacaine (266 mg + 75 mg of group B vs 266 mg of group A) administered. Factors attributable to the reduction of opioid use in the optimized cohort include switching to PRN tramadol and achieving better pain control together with others less readily quantifiable such as changing attitudes of patients in avoiding opioid for postoperative pain, timely accurate assessment of pain, and the adherence to opioid dispensing guidelines by the nursing staff. Other contributing factors of protocol success included complete compliance by the surgical personnel and particularly the dedication of our in-house Advanced Practical Registered Nurses working as the essential link between different care teams to ensure seamless postoperative in-hospital and postdischarge recovery. These factors are difficult to quantify, and they synergize to impact reduction of pain, less opioid use, early discharge, and the overall success of the protocol. It is surprising that pain on day of operation (POD 0) was not affected by mixing bupivacaine with LipoB. We intended to achieve an accelerated onset of intercostal nerve analgesia by co-administering bupivacaine and LipoB as an admixture at 1:2 ratio as recommended by the manufacturer. It is possible that such mixing would affect the pharmacokinetic/pharmacodynamic of the free bupivacaine fraction and reduce its tissue concentration necessary for its immediate anesthetic effects.^13^ Moreover, the medical and social impact of satisfactory postoperative acute pain control cannot be overstated. Early postoperative pain intensity was shown to be a predictor of chronic pain and persistent opioid use.^14^^,^^15^ Prolonged opioid exposure has been linked to reduced survival after lung cancer resection.^16^ Finally, reducing postdischarge narcotics prescription minimizes opioid availability of addiction-prone potent opioids to the public and contributes to the fight against opioid abuse epidemic.

Our overall mortality rate of 0.41% for minimally invasive anatomic pulmonary resections (3/737) is lower than the 0.81% reported for similar national cohort reported by Tong and colleagues.^17^ The major morbidities defined by the Society of Thoracic Surgeons General Thoracic Surgery Database are the equivalent of Clavien-Dindo levels 3 and 4. We observed a major morbidity rate of 5.3% (39/737), which is similar to the recently reported rate of 6.4%.^16^ Moreover, we also observed a greater incidence of MS-DRG 163 and 164 with greater case-mix severity indices in group B. This is most likely attributable to more complete documentation of preoperative comorbidities and in-hospital courses by clinicians and more accurate coding by hospital billing and data management department in more recent years (Tanira Fereira, MD, and Ameena Sherestha, personal spoken and email internal communications 2024). Similar to previous reports,^6^^,^^11^ we observed a net 0.8 day reduction of overall LOS after protocol optimization in the unmatched cohorts (Tables E3 and E4), translating to about 440 extra days available for patients needing robotic anatomic resections (average LOS of 2.6 days) or approximately 169 cases over 4 years. The variances of observed and MS-DRG−expected LOS enabled us to estimate cost-savings (not total hospital costs nor profit margins) due to earlier discharges. It can be viewed as a metric of efficiency from the perspective of the health system (greater safe turnover of beds) and of the third-party paper (less expensive for similar high-quality academic level of care). The overall US$ 2600 cost-savings in group B was very comparable with an average direct and indirect cost reduction of US$ 2800 (robotic lobectomy) by Merritt and colleagues^11^ of Ohio State University or a net reduction of median admission cost of $US 5800 (minimally invasive thoracic procedures) by Young and colleagues^6^ of the University of Virginia.

Our study has many limitations. It is a retrospective single-institution observational case-control study spanning over 6-year period with biases inherent to this type of study. PSM was performed to minimize the impacts of confounders related time-dependent incremental improvement of patient care independent of ERATS. It addressed only anatomic lung resections by robotic approach at an institution deeply ingrained in the ERATS culture by 2 surgeons completely committed to such practice. As such, our findings may not be readily generalizable. The cost-saving analysis was performed only to quantify the impact of shortening of LOS and not a realizable analysis of cost-efficiency of ERATS protocols. Finally, this study was designed to examine the impact of protocol optimization on relevant outcome metrics and not the impact of ERATS over pre-ERATS population. Such a study could not be done because many granular data required for such analysis are not available before the implementation of EPIC in 2018.

## Conclusions

Optimization of an already-functional and successful ERATS protocol is associated with further improvement of postoperative outcomes that remain sustainable over a 4-year study period for patients undergoing robotic pulmonary anatomic resections. This highlights the importance of periodic auditing of the protocol and making modifications for better outcomes and to ensure protocol endurance.

### Webcast

You can watch a Webcast of this AATS meeting presentation by going to: https://www.aats.org/resources/durable-superior-postoperative-9782.

**Figure:**
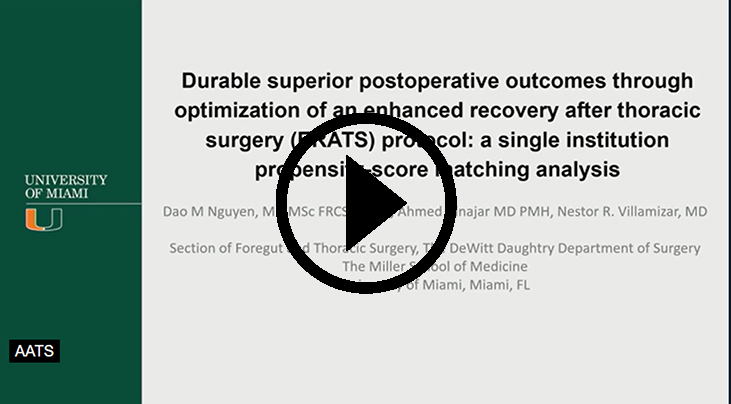

## Conflict of Interest Statement

The authors reported no conflicts of interest. The authors discuss herein the off-label use of liposomal bupivacaine (LipoB) (Exparel, Pacira Pharmaceuticals Inc) for intercostal nerve block, used with institutional approval.

The *Journal* policy requires editors and reviewers to disclose conflicts of interest and to decline handling or reviewing manuscripts for which they may have a conflict of interest. The editors and reviewers of this article have no conflicts of interest.

## Figures and Tables

**Table E1:** Components of ERATS protocol at the University of Miami∗

Preoperative consultation
Extensive counseling of patients and family members about operative plans
Realistic expectation of postoperative recovery and multimodal pain management, manifestation of neuropathic pain and its management
Printed information booklet with instructions
Preoperative clinic visit
Complete review of medical and anesthesia history
Preoperative clearance
Routine preoperative instructions
Perioperative care
Acetaminophen: 1000 mg (1 h before surgery)
Gabapentin: 100 mg (1 h before surgery)
Prophylactic antibiotics (cefazolin 2 g for <120 kg or 3 g > 120 kg; vancomycin 1000 mg for penicillin allergy)
Anesthesia care: Patient-directed fluid management, prophylaxis for postoperative nausea-vomiting (PONV)
**Intercostal nerve blocks and infiltration of surgical wounds with local anesthetics (30 mL of 0.25% bupivacaine admixed with 20 mL of liposomal bupivacaine); 30 mL is used for subpleural infiltration of 2nd-10th intercostal spaces and 20 mL is used to infiltrate the port access before skin incisions and any left-over at the end of skin closure.**
Postoperative care
Analgesics
Acetaminophen 1000 mg PO q8h
**Tramadol 50 mg PO q6h PRN (pain scale <4)**
**Ibuprofen 600 mg PO q8h postoperatively or Toradol 15 mg q6h IV PRN for 2 d (if no medical contraindications); replaced by celecoxib 200 mg q12h starting February 1, 2022; timing of first dose is at the discretion of the attending surgeon**
Gabapentin 100 mg PO q8h; titrating greater doses on the basis of tolerance to achieve control of neuropathic pain
Oxycodone 5 mg PO q6h PRN (pain scale: 4-6)
Oxycodone 10 mg PO q6h PRN (pain scale: 7-10)
Morphine 2 to 4 mg IV q6h PRN or Hydromorphone 0.5-1.0 mg IV or 2-4 mg PO q6h PRN for breakthrough pain
Heparin 5000 U subcutaneous q8h
Metoprolol starting at 12.5 mg q12h (if not already on a beta-blocker following anatomic resection), titrating higher to keep pulse below 80 beats/min. Aggressive management of postoperative new-onset atrial fibrillation with intravenous metoprolol boluses of 5 mg each until rate control or conversion as tolerated without symptomatic hypotension.
Tamsulosin 0.4 mg QD (>50 y old)
Bowel regimen (Colace and Dulcolax scheduled; MiraLAX and milk of magnesia PRN)
Incentive spirometer and ambulation on POD 0
**Regular diet as tolerated on POD 0; high-fat ice cream to rule out chylothorax on POD0**
**Assessment of home oxygen requirement (to prevent discharge delays) by checking for oxygen saturation ≤90% on room air with ambulation**
**Chest tube removal (no air leaks off 20 cm of water suction on postoperative day 1, drainage is not sanguineous, 0.1 to 0.3 mL/kg/h for 6 h on day of tube removal)**
**Foley catheter removal (POD 1) with urine output of at least 1 mL/kg/h overnight of POD 0**
**Intravenous fluid 1 mL/kg until first voiding after removal of Foley catheter**
Frequent pain assessment by nursing staff and APRN and appropriate administration of scheduled and PRN analgesics based on pain level; documentations of pain levels q4h to q6h using then visual analog pain scale.
Discharge plan
Verbal and printed discharge instructions regarding prescriptions, pain management especially signs and symptoms and therapy for neurogenic pain
APRN telephone follow-up POD 3 and POD 7
Contact ARNP or physician's office for advice and management of excessive neuropathic pain
Postdischarge analgesics
Acetaminophen 1000 mg PO q8h for 20 days
**Tramadol 50 mg PO q6h for 3 days (12 tablets, if used postoperatively in-hospital)**
Gabapentin 100 mg PO q8h for 60 days (30 days' supply refill × 1); titrated up to address neurogenic pain
Ibuprofen 600 mg PO q8h or Celecoxib 200 mg q12h for 20 days
** Oxycodone 5 mg or 10 mg PO q6h PRN for 3 days (12 tablets; if used postoperatively in-hospital)**
Pantoprazole 40 mg PO daily for 20 days
Lidocaine patch 4% applied to affected area twice daily PRN
Scheduled 2 and schedule 4 opioid prescriptions are given on the basis of pain levels and the type of opioid use on the day of discharge

*PO*, Per os; *IV*, intravenous; *PRN*, pro re nata; *QD*, quaque die - once a day; *POD*, postoperative day; *APRN*, Advanced Practice Registered Nurse.

∗ Modifications for protocol optimization are highlighted in bold.

**Table E2:** Demographics and clinical characteristics stratified by protocol type

Characteristic	Unmatched, n = 732		Matched, n = 499		
	Optimized ERATS n = 553	Original ERATS n = 179	Optimized ERATS n = 326	Original ERATS n = 173	SMD
Age, y, median (Q1-Q3)	68 (61, 75)	69 (62, 75)	69 (61, 75)	69 (62, 75)	.03
Gender, n (%)					.02
Female	332 (60%)	105 (59%)	196 (60%)	102 (59%)	
Male	221 (40%)	74 (41%)	130 (40%)	71 (41%)	
Race					.19
White	483 (87%)	160 (89%)	287 (88%)	154 (89%)	
Black	35 (6.3%)	12 (6.7%)	17 (5.2%)	12 (6.9%)	
Asian	13 (2.4%)	5 (2.8%)	9 (2.8%)	5 (2.9%)	
Other	22 (4.0%)	2 (1.1%)	13 (4.0%)	2 (1.2%)	
Ethnicity					−.14
Hispanic	262 (47.4%)	81 (45.3%)	156 (47.8%)	79 (46.7%)	
Non-Hispanic	291 (52.6%)	98 (54.7%)	179 (52.2%)	94 (54.3%)	
Hypertension	302 (55%)	103 (58%)	189 (58%)	100 (58%)	.00
Coronary disease	62 (11%)	17 (9.5%)	28 (8.6%)	17 (9.8%)	−.04
Renal failure	5 (0.9%)	3 (1.7%)	2 (0.6%)	0 (0%)	.11
Diabetes mellitus	102 (18%)	31 (17%)	66 (20%)	31 (18%)	.06
Smoking status	344 (62%)	128 (72%)	231 (71%)	122 (71%)	.01
FEV1 (% normal)	88 (76, 100)	88 (76, 97)	86 (74, 99)	88 (76, 97)	.01
DLCO (% normal)	78 (67, 89)	78 (69, 92)	78 (67, 90)	78 (69, 91)	.00

*SMD*, Standardized mean difference; *ERATS*, Enhanced Recovery After Thoracic Surgery; *Q1*, first quartile; *Q3*, third quartile; *FEV1*, forced expiratory volume in 1 second; *DLCO*, diffusing capacity of the lung for carbon monoxide.

**Table E3:** Demographics and clinical characteristics of original ERATS (group A and optimized ERATS (group B) before PSM

Demographics	Group A (n = 184)	Group B (n = 557)	*P* value
Gender, F/M	107/77	332/225	.73
Age, y	69.0 [62.0-75.0]	68.0 [61.0-75.0]	
Race/ethnicity			
White/Black/Asian + other	163 (88.6%)/14 (7.6%)/7 (3.8%)	506 (90.8%)/36 (6.5%)/15 (2.7%)	.58
Hispanic/non-Hispanic	83 (45.1%)/101 (54.9%)	264 (47.4%)/293 (52.6%)	.93
BMI, kg/m^2^	26.3 [23.5-30.2]	27.1 [23.7-30.7]	.22
FEV1 (% N)	87.5 [75.2-98.0]	88.0 [76.0-101.0]	.26
DLCO (% N)	80.0 [68.5-93.0]	78.0 [67.0-91.0]	.37
Intraoperative characteristics			
Operating time, min, median [IQR]	**194.5 [170.7-219.5]**	**180.0 [152.0-223.7]**	**.0005**
Estimated blood loss, mL	**50.0 [50.0-100.0]**	50.0 [30.0-100.0]	**.0002**
Conversion to open	2 (1.1%)	2 (0.4%)	.24
Lobectomy	139	393	.19
Segmentectomy	45	164	
Diagnosis, n (%)			
Benign	7 (3.8%)	24 (4.3%)	.77
Malignant	177 (96.2%)	533 (95.7%)	
Secondary	11 (7.3%)	25 (4.7%)	
Primary	166 (92.7%)	508 (95.3%)	.42
Stage 0-1	**137 (82.5%)**	**373 (73.4%)**	**.017**
Stage 2-4	29 (17.5%)	135 (24.3%)	

Values in bold indicate statistical significance. *F*, Female; *M*, male; *BMI*, body mass index; *FEV1*, forced expiratory volume in 1 second; *DLCO*, diffusing capacity of the lung for carbon monoxide; *IQR*, interquartile range.

**Table E4:** Postoperative outcomes of original ERATS (group A) and optimized ERATS (group B) before PSM

	Group A (n = 182)	Group B (n = 555)	*P* value
Observed LOS, median [IQR]/mean ± SD	**3.0 [2.0-4.0]/3.42 ± 2.62**	**2.0 [1.0-3.0]/2.63 ± 2.73**	**<.0001**
Discharge on POD 1, n (%)	**10 (5.5%)**	**194 (34.9%)**	**<.00001**
Readmission, n (%)	7 (3.8%)	24 (4.3%)	.78
30-d complications (Clavien-Dindo)			
0 (n, %)	**145 (79.67%)**	481 (86.67%)	**.022**
1-2	25 (13.74%)	44 (7.93%)	1-2 vs 3-5: *P* = .53
3-4	11 (6.04%)	28 (5.04%)	
5 (mortality)	1 (0.55%)	2 (0.36%)	
Pain (0-10 visual scale), median [IQR]	n = 179	n = 553	
POD 0	4.8 [3.9-6.2]	4.6 [3.3-5.7]	.142
POD 1	**3.6 [2.0-5.0]**	**2.3 [1.2-3.7]**	**<.0001**
On day of discharge	**2.0 [1.0-3.2]**	**1.3 [0-2.8]**	**<.0001**
Opioid use (MME), median [IQR]			
Average in-hospital	**21.2 [15.0-29.0]**	**12.5 [5.0-25.0]**	**<.0001**
Total in-hospital	**40.0 [25.5-68.0]**	**24.0 [9.5-48.2]**	**<.0001**
30-d postdischarge	**150.0 [60.0-250.0]**	**0 [0.0-60.0]**	**<.0001**
Total postoperative (in-hospital + 30-d postdischarge)	192.0 [115.5-329.3]	**35.5 [10.0-96.5]**	**<.0001**
MME0 (no opioid Rx filling ≤30 d postdischarge)	**22 (12.1%)**	**355 (63.8%)**	**<.0001**

Values in bold indicate statistical significance. *ERATS*, Enhanced Recovery After Thoracic Surgery; *PSM*, propensity-score matching; *IQR*, interquartile range; *SD*, standard deviation; *POD*, postoperative day; *MME*, morphine milligram equivalents.

**Table E5:** Cost-saving analysis of 2 cohorts before propensity score matching

	Group A	Group B	*P* value
**Whole group**	n = 179	n = 553	
Observed LOS, mean ± SD	**3.42 ± 2.62**	**2.63 ± 2.73**	**<.0001**∗
Expected LOS (MS-DRG)	4.53 ± 2.51	5.33 ± 2.45	
Variant expected-observed LOS	0.93 ± 2.40	2.45 ± 3.18	**<.0001**∗
Cost-savings, mean ± SD/median [IQR]	1020.11 ± 2641.00/1100.00 [0.00-2200.00]	2699.28 ± 3497.70/2200.00 [1100.00-4400.00]	**<.0001**†
**DRG 163**	n = 11	n = 57	
Observed LOS, mean ± SD	8.09 ± 4.72	4.44 ± 3.81	**.022**∗
Expected LOS (MS-DRG)	11.70	11.70	
Variant expected-observed LOS	4.40 ± 4.67	7.56 ± 3.81	**.022**∗
Cost-savings, mean ± SD/median [IQR]	**4840.00 ± 5138.57/5500[1100.00-9900.00]**	**8317.54 ± 4188.77/9900.00 [7700.00-11,000.00]**	**.022**†
**DRG 164**	n = 71	n = 310	
Observed LOS, mean ± SD	3.83 ± 2.74	2.88 ± 3.00	**<.0001**∗
Expected LOS (MS-DRG)	5.40	5.40	
Variant expected-observed LOS	1.17 ± 2.74	2.12 ± 3.00	**<.0001**∗
Cost-savings, mean ± SD/median [IQR]	**1285.92 ± 3009.52/2200.00 [1100.00-3300.00]**	**2327.74 ± 3302.86/3300.00 [2200.00-4400.00]**	**<.0001**†
**DRG 165**	n = 92	n = 163	
Observed LOS, mean ± SD	**2.60 ± 1.28**	**1.66 ± 0.83**	**.0021**∗
Expected LOS (MS-DRG)	3.10	3.10	
Variant expected-observed LOS	**0.40 ± 1.28**	**1.34 ± 0.83**	**<.0001**†
Cost-savings, mean ± SD/median [IQR]	**442.39 ± 1412.83** **1100.00 [0.00-1100.00]**	**1477.91 ± 909.55** **2200.00 [1100.00-2200.00]**	**<.0001**†

Values in bold indicate statistical significance. *LOS*, Length of stay; *SD*, standard deviation; *MS-DRG*, Medicare Severity-Diagnosis Related Group; *IQR*, interquartile range.

∗ Student *t* test.

† Mann-Whitney *U* test.
